# Clinical Usefulness of Retropulsion Tests in Persons with Mild to Moderate Parkinson’s Disease

**DOI:** 10.3390/ijerph182312325

**Published:** 2021-11-24

**Authors:** Beata Lindholm, Erika Franzén, Wojciech Duzynski, Per Odin, Peter Hagell

**Affiliations:** 1 Department of Clinical Sciences, Clinical Memory Research Unit, Lund University, 205 02 Malmö, Sweden; 2 Department of Neurology, Rehabilitation Medicine, Memory Disorders and Geriatrics, Skåne University Hospital, 205 02 Malmö, Sweden; Wojciech.Duzynski@skane.se (W.D.); Per.Odin@med.lu.se (P.O.); 3 Division of Physiotherapy, Department of Neurobiology, Care Sciences and Society, 171 77 Stockholm, Sweden; erika.franzen@ki.se; 4 Women’s Health and Allied Health Professionals Theme, Medical Unit Occupational Therapy and Physical Therapy, Karolinska University Hospital, 141 86 Stockholm, Sweden; 5 Division of Neurology, Department of Clinical Sciences Lund, Restorative Parkinson Unit, Lund University, 221 84 Lund, Sweden; Peter.Hagell@hkr.se; 6 The PRO-CARE Group, Faculty of Health Sciences, Kristianstad University, 291 39 Kristianstad, Sweden

**Keywords:** Parkinson’s disease, postural stability, retropulsion, falls, near falls, prediction, pull test, predictive value, sensitivity, specificity

## Abstract

People with Parkinson’s disease (PwPD) have an increased risk for falls and near falls. They have particular difficulties with maintaining balance against an external perturbation, and several retropulsion tests exist. The Unified PD Rating Scale item 30 (UPDRS30) is the most common, involving an expected shoulder pull. Others recommend using an unexpected shoulder pull, e.g., the Nutt Retropulsion Test (NRT). We aimed to evaluate the clinical usefulness of these tests for detecting future fallers. By using two different golden standards related to self-reported prospective falls and near falls over 6 months following two different time points with 3.5 years between, we estimated sensitivity/specificity, Youden index, predictive values, and likelihood ratios for each test. The different time points yielded a different prevalence of falls and near falls, as well as different predictive values. When comparing the performance of the NRT and UPDRS30 for detecting future fallers, we found that the NRT consistently performed better than UPDRS30. However, neither test exhibited optimal performance in terms of predictive values and associated likelihood ratios. Our findings speak against using either of these tests as a single assessment for this purpose and support previous recommendations of using a multifactorial approach when targeting balance problems in PwPD.

## 1. Introduction

Postural instability, i.e., impaired reactive balance, is one of the four cardinal features of Parkinson’s disease (PD). Falls and postural instability are common already in the early stages of PD [1,2] and increase with disease progression [3]. The prevalence of falls and near falls varies between 31–90% [1,2,3] and 26–75% [4,5,6,7], respectively. Transfers and activities in everyday life, such as transferring to/from sitting, walking, and turning, induce self-generated perturbations that challenge reactive balance responses [8] and predispose people with PD (PwPD) to falls [9,10,11]. Walking is particularly challenging since the body is in a state of imbalance, and the only way to prevent falling is to take the next step [12]. Passing over a carpet or transitioning between tiled and wooden surfaces have been identified as the most common environmental factors for falls indoors among PwPD and tripping and slipping are the most common environmental factors outdoors. Importantly, postural instability is the most common individual risk factor for both indoor and outdoor falls [11].

PwPD have particular difficulties in maintaining their balance when being externally perturbed, such as a push backward [13,14,15,16]. This is commonly assessed with retropulsion tests such as an external backward push to challenge their balance responses [13,14]. Item 30 of the Unified Parkinson’s Disease Rating Scale (UPDRS30) is the most common retropulsion test and involves an expected shoulder pull backward [17]. However, others recommend using an unexpected shoulder pull backward, e.g., the Nutt Retropulsion Test (NRT) [14]. These tests are commonly used in clinical settings to indicate risk of falling. Although postural instability is a major influence on falls in PwPD [11], only a few studies have evaluated the clinical usefulness of UPDRS30 and the NRT in terms of their ability to predict falls among PwPD [14,15,18]. For example, Visser et al. [14] evaluated an unexpected shoulder pull according to the NRT and an expected shoulder pull according to UPDRS30 in the same sample. Because of a lack of consensus regarding how an “abnormal” reaction to a shoulder pull should be defined, Visser et al. [14] considered flawless test performance as a normal score, whereas all deviant scorings, regardless of the degree of postural instability, were considered abnormal. However, neither falls nor near falls were recorded prospectively, thus prohibiting the assessment of the predictive value of these tests.

In general, the usefulness of a clinical test in terms of its ability to detect persons with and without the outcome of interest is usually described in terms of sensitivity, specificity, positive predictive value, and negative predictive value [19,20]. High values of both sensitivity and specificity are usually preferred. Sensitivity and specificity are estimated by using an independent confirmation of test results, a so-called gold standard that per definition is unavailable in clinical practice. Therefore, these values are of little practical use when it comes to helping the clinician estimate the probability of the outcome that we seek for individual patients. For this purpose, predictive values may be used, as they estimate the probability of the sought outcome. However, both positive and negative predictive values vary according to the prevalence of the outcome in the population. Therefore, predictive values determined for one population may not be applicable to another, and evaluation of predictive values in various populations is recommended [21,22].

Here we aimed to evaluate the sensitivity, specificity, as well as the positive and negative predictive values of UPDRS30 and the NRT in detecting prospective falls and near falls among persons with mild to moderate PD.

## 2. Materials and Methods

### 2.1. Ethics Statement

All subjects gave their informed consent for inclusion before they participated in the study. The study was conducted in accordance with the Declaration of Helsinki, and the protocol was approved by the Regional Ethics Committee of Lund, Sweden (Dnr 2011/768).

### 2.2. Study Design

This prospective cohort study is a part of a larger project [5], including two time points (T1 and T2) with a first assessment (T1) at the start of the study and a second assessment (T2) 3.5 years later. Each assessment was followed by the prospective recording of falls and near falls during the subsequent 6 months.

### 2.3. Participants

All PwPD [23] who received care at a neurology outpatient clinic in the south of Sweden were considered eligible for inclusion in the study (*n* = 359). Exclusion criteria at the start of the study were age above 80 years old (*n* = 121), inability to stand without support (i.e., spontaneous tendency to fall in a standing position; *n* = 22), unable to understand instructions (*n* = 14), and severe comorbidity (*n* = 11). Of the remaining 191 potential participants, 40 (16 women) declined participation. Of 151 PwPD at T1, five did not complete the prospective assessment of falls and near falls. The final study sample at T1, therefore, included 146 PwPD.

At T2, 24 PwPD dropped out due to severe comorbidity or death, and 49 (17 women) declined participation. Severe comorbidities included metastatic cancer, stroke, heart failure, dialysis-treated renal failure, age-related eye diseases, gastrointestinal diseases, and diabetic complications. PwPD with an inability to stand without support were not excluded at T2. Of the remaining 73 PwPD at T2, an additional 15 did not complete the prospective assessment of falls and near falls, leaving data from a total of 58 PwPD available for analyses.

### 2.4. Assessments and Procedure

Detailed descriptions of the procedures are available elsewhere [5]. Clinical assessments were administered by the same physical therapist with extensive experience of movement disorders (BL). At T1 and T2, all participants were assessed during outpatient visits, which were scheduled at a time of day when they reported that they usually felt at their best. All participants self-rated their motor status at the time of examination as “good/on,” “on with dyskinesia,” or ”bad/off,”

Retropulsion was assessed using an unexpected shoulder pull according to the NRT [13] as well as an expected shoulder pull according to UPDRS30 [17]. The participant was standing with her/his feet slightly apart and eyes open, with the examiner giving a sudden, firm backward pull to the shoulders from behind. The NRT was executed first and scored 0–3: 0 (normal, ≤2 steps to recover), 1 (≥3 or more steps; recovers unaided), 2 (would fall if not caught), 3 (spontaneous tendency to fall or unable to stand unaided) [13,14]. This was followed by a 10-Meter Walk Test in comfortable gait speed (for another study questions, see [5]). Motor symptoms were assessed using the UPDRS part III (0–108, higher = worse) [17], and PD severity was assessed according to Hoehn and Yahr (HY) (I–V, higher = worse) [24]. Cognition was evaluated using the Mini-Mental State Examination (MMSE) (0–30, higher = better) [25]. During assessments according to UPDRS30 (as part of the UPDRS part III), the participant was first told that s/he was to be pulled and instructed to prevent falling [17,26]. Performance was scored 0–4: 0 (normal), 1 (retropulsion, but recovers unaided), 2 (absence of postural response, would fall if not caught by the examiner), 3 (very unstable, tends to lose balance spontaneously), and 4 (unable to stand without assistance).

As the last step during each outpatient visit, the definitions of a fall and a near fall were thoroughly described to participants. Falls were defined as “an unexpected event in which the person comes to rest on the ground, floor, or lower level” [27]. Near falls were defined as ”a fall initiated but arrested by support from the wall, railing, other person, etc.” [28]. Participants were then provided with a diary for the prospective recording of falls and near falls. This included the date and time of every fall/near fall event and an indication of whether it was a fall or a near fall. The question in relation to a fall was phrased as follows: Did you fall in such a way that your body hit the ground? The corresponding question about a near fall incident was phrased: Were you close to falling, but managed to brace yourself at the last moment (e.g., grabbed on to someone, to an object or the wall)? Both questions were answered by Yes or No. After the study visits, all participants were telephoned monthly to ensure that registrations of falls and near falls had been completed according to instructions. During the last telephone call, they were asked to return the diary in a pre-stamped envelope.

Data on demographics (age, gender, PD duration) and anti-parkinsonian medications were obtained from medical records. Daily levodopa equivalent (LDE) doses (mg/day) were calculated according to recommended conversion factors [29].

### 2.5. Statistical Analysis

Data were checked regarding underlying assumptions and analyzed accordingly using SPSS version 27 (IBM Corp., Armonk, NY, USA) and WinPepi version 11.65 [30]. Normally distributed interval/ratio level variables were described using means and SDs. In other cases, n (%) and median (q1–q3) were used as appropriate. The alpha level of significance was set at 0.05. Group comparison between those at T1 who dropped out at T2 (*n* = 88) and those at T1 who also participated at T2 (*n* = 58) was performed using the Mann-Whitney U test for ordinal or continuous variables and Chi-square test for categorical variables. Receiver operating characteristics (ROC) curve analysis was used to determine the sensitivity and specificity for each possible cut-off score of the NRT and UPDRS30 [19]. Sensitivity is the probability that a patient has a positive test result given that they have a positive outcome, while specificity is the probability that a patient has a negative test result given that they have a negative outcome. Two different outcomes (gold standards) collected during the prospective monitoring of falls and near falls were used in the analyses: (i) at least one reported fall and (ii) at least one reported fall and/or near fall (i.e., including those reporting both) [5]. The Youden index (sensitivity + specificity − 1), which may range between 0 and 1 (the larger, the better) [19], was estimated for each possible score of the NRT and UPDRS30. The cut-off score associated with the highest Youden index was chosen as the optimal cut-off score to discriminate between PwPD with and without the respective outcomes. Maximizing sensitivity and specificity minimize false positive and false negative identifications.

Based on the sensitivity and specificity, we also calculated the ratio of the probability of a positive test result if the outcome is positive (true positive) to the probability of a positive test result if the outcome is negative (false positive), i.e., the likelihood ratio of a positive test result (LR+). LR+ represents the increase in odds favoring the outcome given a positive test result. Similarly, we also calculated the ratio of the probability of a negative test result if the outcome is positive to the probability of a negative test result if the outcome is negative (LR−), which represents the increase in odds favoring the outcome given a negative test result [19].

The positive predictive value (PPV) and negative predictive value (NPV) were also calculated. The PPV is the probability of a positive outcome given a positive test result. This contrasts with sensitivity, which is the probability of a positive test result given a positive outcome. Similarly, the NPV is the probability of a negative outcome given a negative test result (differing from specificity, which is the probability of a negative test result given a negative outcome). In general, the higher the prevalence of the sought outcome, the less useful NPV is, while the response to PPV becomes more valuable. With decreasing prevalence, the reverse is true [19].

The clinical usefulness of the test is regarded as high if PPV and LR+ are high and if NPV is high and LR− is low [19].

## 3. Results

At T1, the sample consisted of 146 PwPD (46% women) with a mean age of 68 (SD, 9.6) years and a mean disease duration of 4 (SD, 3.9) years. Their median (q1–q3) UPDRS III score and HY stage was 12 (8–18) and 2 (2–3), respectively. The median (q1–q3) scores for both the NRT and UPDRS30 were 0 (0–1). T2 included 58 PwPD (55% women) with a mean age of 69 (8.7) years and a mean disease duration of 7 (3.7) years. Their median (q1–q3) UPDRS III score and HY stage was 13.5 (9–20) and 2 (2–3), respectively. The median (q1–q3) NRT and UPDRS30 scores were both 0 (0–1). Further details are provided in Table 1.

The sample who dropped out at T2 consisted of 88 PwPD (42% women) with a mean age of 69 (SD, 2.14) years and PD duration of 4.6 (SD, 4.5) years at T1. To compare with those who participated at both T1 and T2, their disease duration was significantly longer (*p* < 0.001) and their self-rated motor status significantly worse (*p* = 0.025). There were no significant differences regarding age, gender, disease severity, motor symptoms, daily total levodopa equivalent doses, cognition, or balance tests (*p* ≥ 0.68). Further details are provided in the Supplementary Materials, Table S1.

The prevalence of at least one fall or at least one fall and/or near fall among 146 PwPD during the prospective T1 monitoring was 32% and 46%, respectively. Results from the ROC curve analyses were significant (*p* < 0.010) for both tests (NRT and UPDRS30) and for both outcomes. For detecting PwPD with/without prospective falls at T1, the highest Youden index (0.32) was found for an NRT cut-off score of 1. The corresponding sensitivity/specificity was 0.47/0.85, PPV/NPV was 0.60/0.77, and LR+/LR− was 3.09/0.63. For detecting PwPD with/without prospective falls and/or near falls at T1, the highest Youden index (0.28) was also found for an NRT cut-off score of 1, with corresponding sensitivity/specificity of 0.40/0.87; PPV/NPV of 0.73/0.77, and LR+/LR− of 3.18/0.68. Further details are provided in Table 2. Corresponding ROC curve analyses for tests results at T1 for the 58 PwPD included in T2 were not significant neither for the NRT nor UPDRS30 (*p* ≥ 0.54).

The prevalence of at least one fall or at least one fall and/or near fall among 58 PwPD during the prospective T2 monitoring was 45% and 52%, respectively. Results from ROC curve analyses were significant (*p* ≤ 0.043) for NRT for both outcomes. Corresponding analyses for UPDRS30 were not significant (*p* ≥ 0.079). Therefore, estimation of sensitivity/specificity and PPV/NPV was not applicable for UPDRS30. For detecting PwPD with/without prospective falls as well as falls and/or near falls at T2, the highest Youden indices (0.26 and 0.23, respectively) were found for the NRT cut-off score of 1. Further details are provided in Table 3.

## 4. Discussion

In this study, we evaluated the clinical usefulness of two commonly used retropulsion tests, the NRT and UPDRS30, for detecting future falls and near falls among persons with mild to moderate PD. By using two different golden standards, i.e., prospectively self-reported falls and falls and/or near falls over 6 months following two different time points with 3.5 years between, we estimated sensitivity/specificity, Youden index, predictive values, and likelihood ratios for each cut-off value and for each test. The different time points yielded different prevalence of falls and near falls, as well as different predictive values. However, when comparing the performance of the NRT and UPDRS30 as indicators of future falls and near falls, we found that the NRT consistently performed better than UPDRS30. Importantly, neither test exhibited optimal performance in terms of predictive values and associated LR [19]. This suggests that whereas an unexpected (i.e., the NRT) is more useful than an expected shoulder pull (i.e., UPDRS30), both retropulsion tests are of limited clinical value in terms of predicting future falls and near falls in PwPD. Although these clinical tests are commonly used, this is not unexpected since falls are multifactorial [31,32,33,34] and not only related to reactive balance responses. Therefore, it is reasonable to suggest that clinical prediction of future falls needs to be based on more than one indicator. For example, the 3-step falls prediction model (including gait speed and history of falls and freezing of gait) has been found to be a quick and useful means of predicting future falls in PwPD [35,36,37]. Moreover, history of falls, abnormal tandem gait, and cognitive impairment have been found to be predictive of the number of falls/near falls in PwPD [38].

Our results regarding the advantage of the NRT over UPDRS30 as a predictor of future falls are in line with those of Visser et al., who also evaluated both tests at the same time [14]. However, their estimates of sensitivity (0.63) and PPV (0.86) indicate better usefulness of NRT compared with that found in our study. This may be explained by differences in the definition of the golden standard, as in addition to falls and near falls, Visser et al. also considered adaptations to prevent falls (i.e., use of walking aids). This extension of the golden standard definition was conducted to reduce the risk that participants with impaired postural stability were inadvertently classified as non-fallers. In another study, Valcovic et al. [18] evaluated the clinical usefulness of the NRT for the prediction of falls. Their reported sensitivity (0.69) and PPV (0.76) were higher than those estimated in our study. This may, however, be due to differences in sample characteristics. That is, the participants in the study by Valcovic et al. [18] had longer disease duration and more severe motor symptoms than our sample. In fact, it has been suggested that retropulsion tests are more useful for the detection of fallers in the later stages of PD due to more severe postural instability [39]. Importantly, considering near falls in defining the golden standard, as in our study, and, thus, focusing on early aspects of postural instability in PwPD [40] contributed to a higher PPV. This is not surprising because the history of near falls has been reported to be a more powerful predictor of future falls than a history of falls [5]. However, monitoring of near falls, based on self-reports as in our study, may underestimate the true prevalence of such events [41,42]. There is ongoing research that aims to develop inertial sensors to improve detection of near falls and evaluation of fall risk [43,44,45].

Jacobs et al. [15] evaluated sensitivity/specificity of several tests such as the Push & Release (P&R) test and UPDRS30 in relation to falls as a golden standard. Reported sensitivity (0.35) and specificity (0.96) of UPDRS30 was largely in line with the results in our study and indicated the poor ability of this test for the detection of fallers. The P&R test, which includes an expected perturbation induced by the patient leaning backward while supported by the examiner, showed similar results (sensitivity 0.41 and specificity 0.77) [15]. Importantly, two comprehensive assessments of postural instability (the Mini-BESTest and Brief-BESTest) outperformed the P&R test and UPDRS30 [15]. Therefore, clinicians may benefit from using multi-item balance tests instead of a single-item when aiming to detect falls in PwPD. Furthermore, multi-item balance tests may have the additional benefit of guiding preventative interventions targeting balance impairments for those individuals who are classified to be at risk for future falls [15].

The major limitations of this study are that at T1, those with an inability to stand without support, i.e., spontaneous tendency to fall in the standing position, were excluded, which was not the case at T2. Moreover, at T2, the most severely affected PwPD, especially men and older persons, declined from participation, which challenges generalizability. Further, most of the participants in this study were relatively mildly affected by PD, and those above the age of 80 years were initially excluded. Thus, our findings may not apply to very old PwPD or those with more severe PD. However, our study samples appear representative of its target population. Furthermore, focusing on PwPD in the relatively early stages of the disease has been recommended for professionals to work proactively in preventing falls [46,47].

## 5. Conclusions

The clinical usefulness of the NRT and UPDRS30 for the prediction of falls and near falls is limited. Our findings speak against using either of these tests as a single assessment alone for this purpose and support previous recommendations of using a multifactorial approach when targeting balance difficulties and falls in PwPD.

## Figures and Tables

**Table 1:** Sample characteristics.

	Time 1 (*n* = 146)	Time 2 (*n* = 58)
Age (years), mean (SD; min–max)	68 (9.6; 35–80)	69 (8.9; 46–84)
Female gender, n (%)	67 (46)	32 (55)
PD duration (years), mean (SD; min–max)	4.0 (3.9; 0.1–17.0)	7.0 (3.7; 3.6–20)
PD severity (HY), median (q1–q3; min–max)	2 (2–3; 1–4)	2 (2–3; 1–4)
Daily total levodopa equivalent (LDE) dose (mg), median (q1–q3; min–max)	400 (300−600; 0–1477)	500 (400–675; 150–1580)
Self-rated motor status at the time of clinical examination “on” or “on with dyskinesias,” n (%) “off,” n (%)	128 (88) 18 (12)	48 (83) 10 (17)
Motor symptoms (UPDRS part III), median (q1–q3; min–max)	12 (8–18; 1–46)	13.5 (9–20; 1–40)
Cognition (MMSE), median (q1–q3; min–max)	28 (26–29; 19–30)	28 (27–29; 20–30)
Retropulsion (NRT), median (q1–q3; min–max)	0 (0–1; 0–2)	0 (0–1; 0–3)
Retropulsion (UPDRS30), median (q1–q3; min–max)	0 (0–1; 0–2)	0 (0–1; 0–3)

HY, Hoehn and Yahr; MMSE, Mini-Mental State Examination; NRT, Nutt Retropulsion Test; PD, Parkinson’s disease; UPDRS, Unified Parkinson’s Disease; Rating Scale; UPDRS part III, motor score of the Unified Parkinson’s Disease Rating Scale; UPDRS30, item 30 of UPDRS.

**Table 2:** Sensitivity, specificity, Youden index, predictive values, and likelihood ratios for different cut-off scores of the NRT and UPDRS30 with prospective falls and near falls as golden standards at Time 1 (*n* = 146).

	*p*-Value for ROC Analysis	Cut-Off Score	Sens (95% CI)	Spec (95% CI)	YI (95% CI)	PPV (95% CI)	NPV (95% CI)	LR+ (95% CI)	LR− (95% CI)
Golden standard: falls (prevalence, 32%)									
NRT (0–3) ^1^	<0.001	1	0.47 (0.33–0.61)	0.85 (0.77–0.10)	0.32 (0.16–0.48)	0.60 (0.46–0.72)	0.77 (0.72–0.82)	3.09 (1.77–5.39)	0.63 (0.47–0.83)
		2	0.34 (0.22–0.48)	0.96 (0.90–0.98)	0.30 (0.16–0.44)	0.80 (0.60–0.91)	0.75 (0.71–0.91)	8.43 (2.98–23.82)	0.69 (0.56–0.82)
UPDRS i 30 (0–4) ^2^	0.010	1	0.51 (0.37–0.65)	0.70 (0.60–0.78)	0.21 (0.04–0.38)	0.44 (0.35–0.55)	0.75 (0.69–0.83)	1.69 (1.12–2.54)	0.70 (0.51–0.97)
		2	0.19 (0.10–0.34)	0.95 (0.89–0.98)	0.14 (0.02–0.26)	0.64 (0.40–0.83)	0.71 (0.68–0.74)	3.79 (1.34–10.69)	0.85 (0.74–0.99)
Golden standard: falls and/or near falls (prevalence, 46%)									
NRT (0–3) ^1^	0.001	1	0.40 (0.29–0.52)	0.87 (0.78–0.93)	0.28 (0.14–0.41)	0.73 (0.59–0.84)	0.63 (0.58–0.68)	3.18 (1.66–5.49)	0.68 (0.55–0.85)
		2	0.25 (0.17–0.37)	0.93 (0.89–0.99)	0.22 (0.10–0.33)	0.85 (0.65–0.95)	0.60 (0.57–0.64)	6.68 (2.05–21.82)	0.78 (0.67–0.90)
UPDRS30 (0–4) ^2^	0.005	1	0.49 (0.38–0.61)	0.73 (0.63–0.82)	0.23 (0.07–0.38)	0.61 (0.50–0.71)	0.63 (0.57–0.69)	1.85 (1.19–2.88)	0.69 (0.53–0.91)
		2	0.15 (0.08–0.25)	0.95(0.88–0.98)	0.10 (0.00–0.20)	0.71 (0.46–0.88)	0.57 (0.54–0.60)	2.95 (0.97–8.97)	0.90 (0.80–1.00)

^1^ those with score 3 were not included at Time 1; ^2^ those with scores 3 or 4 were not included at Time 1; YI, Youden index; LR+/−, positive/negative likelihood ratio; NPV, negative predictive value; NRT, Nutt Retropulsion Test; PPV, positive predictive value; ROC, receiver operating characteristics; Sens, sensitivity; Spec, specificity; UPDRS30, item 30 of the Unified Parkinson’s Disease Rating Scale.

**Table 3:** Sensitivity, specificity, Youden index, predictive values, and likelihood ratios for different cut-off scores of the NRT and UPDRS30 with prospective falls and near falls as golden standards at Time 2 (*n* = 58).

	*p*-Value for ROC Analysis	Cut–Off Score	Sens (95% CI)	Spec (95% CI)	YI (95% CI)	PPV (95% CI)	NPV (95% CI)	LR+ (95% CI)	LR− (95% CI)
Golden standard: falls (prevalence, 45%)									
NRT (0–3)	0.034	1	0.48 (0.30–0.67)	0.78 (0.61–0.89)	0.26 (0.02–0.50)	0.63 (0.46–0.79)	0.66 (0.56–0.75)	2.19 (1.01–4.75)	0.67 (0.44–1.01)
		2	0.23 (0.11–0.42)	0.91 (0.77–0.98)	0.14 (−0.05–0.33)	0.68 (0.28–0.95)	0.59 (0.53–0.65)	2.46 (0.68–8.90)	0.85 (0.67–1.08)
		3	0.08 (0.02–0.24)	1.00 (0.89–1.00)	0.08 (−0.03–0.18)	1.00 (0.13–1.00)	0.57 (0.52–0.62)	NA (0.18–NA)	0.92 (0.83–1.03)
UPDRS30 (0–4)	0.079	NA							
Golden standard: falls and/or near falls (prevalence, 52%)									
NRT (0–3)	0.043	1	0.45 (0.28–0.63)	0.79 (0.61–0.90)	0.23 (0.00–0.47)	0.68 (0.50–0.83)	0.58 (0.48–0.67)	2.09 (0.92–4.73)	0.70 (0.48–4.73)
		2	0.20 (0.10–0.37)	0.89 (0.73–0.96)	0.09 (−0.09–0.28)	0.66 (0.27–0.91)	0.51 (0.45–0.57)	1.87 (0.52–6.76)	0.90 (0.72–1.12)
		3	0.07 (0.02–0.21)	1.00 (0.88–1.00)	0.07 (−0.02–0.16)	1.00 (0.15–1.00)	0.50 (0.45–0.56)	NA (0.16–NA)	0.93 (0.85–1.03)
UPDRS30 (0–4)	0.231	NA							

YI, Youden index; LR+/−, positive/negative likelihood ratio; NA, not applicable; NPV, negative predictive value; NRT, Nutt Retropulsion Test; PPV, positive predictive value; ROC, receiver operating characteristics; Sens, sensitivity; Spec, specificity; UPDRS30, item 30 of the Unified Parkinson’s Disease Rating Scale.

## Data Availability

The datasets generated during and/or analysed during the current study are not publicly available due to Swedish and EU personal data legislation but are available from the corresponding author on reasonable request. Any sharing of data will be regulated via a data transfer and user agreement with the recipient.

## Supplementary Materials

The following are available online at https://www.mdpi.com/article/10.3390/ijerph182312325/s1, Table S1: comparison between those at T1 who dropped out at T2 (*n* = 88) and those at T1 who participated at T2 (*n* = 58).
