# Supplementary material for: Clinical Usefulness of Retropulsion Tests in Persons with Mild to Moderate Parkinson’s Disease
Source: Int J Environ Res Public Health. 2021 Nov 24;18(23):12325. doi: 10.3390/ijerph182312325 (PMC8656501; doi:10.3390/ijerph182312325)
Supplement: Supplementary file 1 [file ijerph-18-12325-s001.zip › ijerph-1420185-supplementary.pdf]

Table S1. Comparison between those at T1 who dropped out at T2 (n=88) and these at T1 who participated at T2 (n=58)

	**Drop out** (n=88)	**Time 1** (n=58)	**P value**
Age (years), mean (SD; min–max)	69 (2.14; 35–80)	65 (8.8; 43-80)	0.570[a]
Female gender, n (%)	37 (42)	32 (55)	0.640[b]
PD duration (years), mean (SD; min–max)	4.6 (4.5; 0.1–15)	3.0 (3.6; 0.1-17)	< 0.001[a]
PD severity (HY), median (q1–q3; min–max)	2 (2–3; 1–4)	2 (2–3; 1–4)	0.771[a]
Daily total levodopa equivalent (LDE) dose (mg), median (q1–q3; min–max)	430 (300–600; 0–1318)	300 (195–566; 0–1477)	0.164[a]
Self–rated motor status at the time of clinical examination			0.025[b]
“on” or “on with dyskinesias”, n (%)	84 (95)	48 (83)	
“off”, n (%)	4 (5)	10 (17)	
Motor symptoms (UPDRS part III), median (q1–q3; min–max)	16 (9–20;19–30)	10 (7–14; 2–28)	0.670[a]
Cognition (MMSE), median (q1–q3; min–max)	28 (26–29; 19–30)	28.5 (27–29; 23–30)	0.317[a]
Retropulsion (NRT), median (q1–q3; min–max)	0 (0–0; 0–2)	0 (0–1; 0–2)	0.280[a]
Retropulsion (UPDRS30), median (q1–q3; min–max)	0 (0–1; 0–2)	0 (0–1; 0–2	0.643[a]

[a]Mann-Whitney U test
[b]Chi-Square test
HY, Hoehn and Yahr; MMSE, Mini–Mental State Examination; NRT, Nutt Retropulsion Test; PD, Parkinson’s disease; UPDRS, Unified Parkinson’s Disease Rating Scale; UPDRS part III, motor score of the Unified Parkinson’s Disease Rating Scale; UPDRS30, Item 30 of UPDRS.
